# SIGNAL-TO-NOISE RATIO RATE MEASUREMENT IN FLUOROSCOPY FOR QUALITY CONTROL AND TEACHING GOOD RADIOLOGICAL IMAGING TECHNIQUE

**DOI:** 10.1093/rpd/ncaa222

**Published:** 2021-02-01

**Authors:** Henrik Elgström, Erik Tesselaar, Michael Sandborg

**Affiliations:** Department of Medical Radiation Physics, Department of Health, Medicine and Caring Sciences, Linköping University, 58185 Linköping, Sweden; Department of Medical Radiation Physics, Department of Health, Medicine and Caring Sciences, Linköping University, 58185 Linköping, Sweden; Department of Medical Radiation Physics, Department of Health, Medicine and Caring Sciences, Linköping University, 58185 Linköping, Sweden; Centre for Medical Image Science and Visualisation (CMIV), Linköping University, 58185 Linköping, Sweden

## Abstract

Visibility of low-contrast details in fluoroscopy and interventional radiology is important. Assessing detail visibility with human observers typically suffers from large observer variances. Objective, quantitative measurement of low-contrast detail visibility using a model observer, such as the square of the signal-to-noise ratio rate (SNR^2^_rate)_, was implemented in MATLAB**™** and evaluated. The expected linear response of SNR^2^_rate_ based on predictions by the so-called Rose model and frame statistics was verified. The uncertainty in the measurement of SNR^2^_rate_ for a fixed imaging geometry was 6% based on 16 repeated measurements. The results show that, as expected, reduced object thickness and x-ray field size substantially improved SNR^2^_rate_/P_KA,rate_ with P_KA,rate_ being the air kerma area product rate. The measurement precision in SNR^2^_rate_/P_KA,rate_ (8–9%) is sufficient to detect small but important improvements, may guide the selection of better imaging settings and provides a tool for teaching good radiological imaging techniques to clinical staff.

## INTRODUCTION

The assessment of the performance of an imaging system is ultimately a measure of the amount of diagnostic information that an operator can derive for a specific task^(1)^. Evaluations of x-ray systems performance must also consider absorbed doses to patients’ organs. Clinical image quality of the imaging system can be evaluated using receiver operating characteristics^(2)^ or visual grading of specific image criteria^(3)^. However, physical image quality indices such as contrast, noise, artifacts and spatial and temporal resolution are more commonly considered in quality control measurement. Favorable characteristics of these indexes should include clinical relevance, reproducibility, accuracy, precision, sensitivity and ease of measurement.

Low-contrast detail detectability is an important image quality index in fluoroscopy and is primarily dependent on contrast, sharpness and background noise. Various methods are used to assess the imaging system’s performance in this respect. For quality control purposes, evaluation of the visibility of various low-contrast details by human observers is common; for example, threshold contrast detection and possibly multiple-alternative forced-choice detection experiments using low-contrast cylinder discs test objects. These experiments are typically limited by problems of subjectivity and lack of precision^(4–6)^. This is because human visual detection is observer-dependent, and it is difficult to define, communicate and maintain a criterion on what is actually visible in a reliable way.

**Figure 1:**
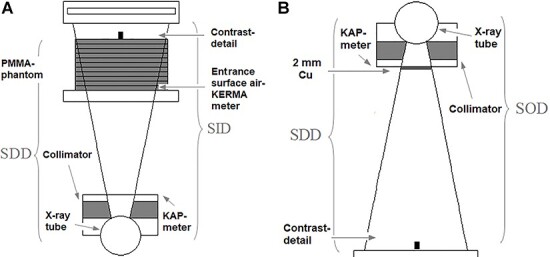
Schematic view of the two measurement geometries: (**a**) FOM (Setup 1) and (**b**) quality control (Setup 2). SDD is source to contrast detail distance and SID is source to image detector distance

SKE/BKE (signal/background known exactly) is the simplest and most ideal task where the target to be detected is fully known and variation in the image data is due to stochastic effects^(7)^. Under these circumstances, a class of objective mathematical ideal model observers, derived from statistical decision theory, can estimate signal-to-noise ratio (SNR) based on the theoretically most efficient use of information. No general correlation between the physical image quality indices and clinical image quality exists^(1,8–10)^. Model observers^(11,12)^ can still fulfill a role in routine quality control of the imaging system performance if most of the favorable characteristics mentioned previously are met. In addition, analysis of the effect of an imaging equipment parameter on SNR and patient dose indices, such as dose rate, mode of operation, imaging geometry, x-ray field size and photon energy, forms a basis for its clinical operation and if properly analyzed, it can be a useful teaching tool for the operator. The ratio between this image quality index and the patient dose index is a figure of merit (FOM) and is here computed as FOM_K_ = SNR^2^_rate_/K_rate_ and FOM_KA_ = SNR^2^_rate_/P_KA,rate_ and sometimes called dose efficiency. Here K_rate_ is the incident air kerma rate at the phantom surface and P_KA,rate_ the air kerma area product rate.

The objectives of this work were to (1) encode and validate the SNR^2^_rate_ model observer software used in FluoroQuality in MATLAB™ to (2) explore the model observer usefulness for quality control on a fluoroscopy unit and to (3) perform measurements of FOM_K_ and FOM_KA_ as a tool for teaching imaging physics to clinical staff and optimizing radiological protection.

## MATERIALS AND METHODS

### Model observer and signal-to-noise rate measurements

In the current study, ideal and quasi-ideal model observers have been used for measurements of the accumulating rate of the square of the SNR, SNR^2^_rate_ of contrast details^(13,14)^ on two fluoroscopy units. The SNR^2^_rate_ detection index is the natural choice as FOM considering the integration of information over time in real-time x-ray viewing. The methods are based on experiments of binary response, which require two hypotheses: H_1_: signal present and H_0_: signal absent. The decision criterion in statistical decision theory is based on the rating of confidence for a decision between the two hypotheses: H_1_ and H_0_. The degree of confidence that a certain image belongs to either H_1_ or H_0_ is quantified by a conditional decision variable (CDV)^(1)^. An assumption according to this theory is that CDVs from the two sets of images under the same imaging conditions will be grouped into one of two normal distributions belonging to each class. Detection performance is therefore expressed in terms of the separation between these two distributions^(1,7)^.

A quasi-ideal DC and high frequency suppressing model observer SNR^2^_rate_^(7)^ was implemented in a MATLAB™ (version 2019a, The MathWorks, Inc, Natick, Massachusetts, USA) code^(15)^. This model observer is constructed from the difference between the mean signal of the image frames (here 900 or 1024) containing a low-contrast detail and the same number of frames in the same part of the image detector without the low-contrast detail. The model observer template is then cross-correlated with each image frame separately with and without the contrast detail to form the observer’s CDVs. Specific image frames analyzed were sequentially removed from the image stack. The remaining images were used to form the observer template, in order to minimize bias.

The SNR of single frame (SNR_sf_) was computed from the average difference and variances of the two conditional distributions: one for signal present and signal absent cases. However, neighboring frames in a sequence are not independent, and hence a lag-factor, F [unit s^−1^], is calculated from the spatial–temporal noise power spectrum to account for the number of independent frames per second such that SNR^2^_rate_ = SNR^2^_sf_^.^ F, for details see Tapiovaara^(7)^.

**Figure 2:**
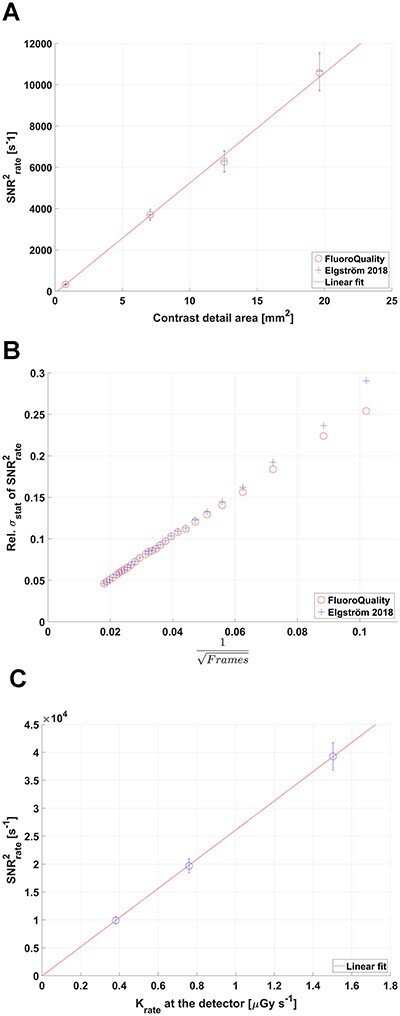
(**a**): Comparison of SNR^2^_rate_ as a function of the area of a 3 mm thick cylindrical aluminum (Al) disc contrast detail using Setup 1 between the original FluoroQuality code (o) (Tapiovaara (2003) and the MATLAB implementation (Elgström 2018) (+). The error bars indicate 1 SD corresponding to 7%. (**b**): The relative statistical uncertainty in SNR^2^_rate_ as function of the inverse square root of the total number of frames used in the analysis; FluoroQuality code (o) and (Elgström 2018) (+). (**c**) SNR^2^_rate_ as a function of air kerma rate at the image detector housing (tube current 10, 20, 40 mA) for an Al disc (4 mm thick and 6 mm diameter) using Setup 2

**Table 1:** Acquisition modes and ADRC, parameters in the experiments with two fluoroscopy imaging systems from Siemens Healthineers.

Parameter	Axiom Artis Zee MP (Setup 1)	Cios Alpha (Setup 2)
Purpose of measurement	FOM	Quality control
Protocol name	Esophagus-Barium	Thorax
Dose mode setting	Medium	Low
Attenuating phantom	PMMA	Cu
Added filtration (mm Cu)	0.3	0.1
Field of view	42	30
Post processing	Enabled	Enabled
Matrix size	1024^2^	768^2^
Frames in analysis	1024	900
Region of interest pixels	64^2^	64^2^
Tube voltage (kV)	81	75
Tube current (mA)	varying	7
SID (cm)	110 or 120	110
SDD (cm)	varying	106
Focal spot size (mm)	0.6	0.3
Pulse length (ms)	3.5–16	5
Pulse rate (s^−1^)	15	15
Contrast detail material	Soft and lung tissue	Al
Contrast detail density (g.cm^−3^)	(see www.cirsinc.com) 1.05 and 0.21	2.7

**Table 2:** Image quality metric, dosimetric indices and figures of merit for different x-ray field size. A 20 cm thick phantom, 81 kV tube voltage, 0.3 mm Cu filtration and constant pulse length but decreasing tube current were used. A low density (lung tissue) 15 mm thick contrast detail was used

X-ray Field size (cm^2^)	SNR^2^_rate_ (s^−1^)	K_rate_ (μGy.s^−1^)	P_KA,rate_ (μGy.m^2^.s^−1^)	SNR^2^_rate_/K_rate_ (μGy^−1^)	SNR^2^_rate_/P_KA,rate_ (μGy^−1^.m^−2^)
236 ± 11	770 ± 62	102 ± 1	0.98 ± 0.01	7.56 ± 0.62	784 ± 64
441 ± 15	695 ± 55	82 ± 1	1.62 ± 0.02	8.50 ± 0.67	429 ± 34
658 ± 18	654 ± 53	78 ± 1	2.21 ± 0.03	8.40 ± 0.68	295 ± 24
870 ± 21	542 ± 46	77 ± 1	2.74 ± 0.03	7.05 ± 0.60	198 ± 17

## Experiments

### Imaging system, instrumentation and measurements of FOM

Images and dosimetric quantities were collected from two Siemens fluoroscopy systems at Linköping University Hospital (Axiom Artis Zee MP and Cios Alpha, Siemens Healthineers, Erlangen, Germany). Images were sent to the picture archiving and communication system (PACS) or saved to a USB-flash drive for further image analysis using FluoroQuality^(7)^ and a validated in-house MATLAB™ code^(15)^. P_KA,rate_ was measured with a transmission ionization chamber built into the collimator assembly (Diamentor KAP meter, PTW, Freiburg, Germany) divided by the fluoroscopy time from the Dicom header information. The readings from the built-in KAP meter were compared with a calibrated Radcal™ PDC (Patient Dose Calibrator, Monrovia, USA) KAP meter and its reading corrected for the attenuation in the patient couch. K_rate_ was measured with a calibrated T20 solid-state detector coupled to a Piranha multipurpose detector (RTI Group, Mölndal, Sweden). Both K_rate_ and P_KA,rate_ were traceable to the Swedish secondary standards laboratory.

Figures of merit with respect to K_rate_ and P_KA,rate_ i.e. FOM_K_ and FOM_KA_ were studied as a function of phantom thickness, source to-object distance and x-ray field size.

### Imaging geometry and imaging parameters

In the FOM measurements, the patient was represented by a stack of homogeneous polymethyl-methacrylate (PMMA) blocks with a surface area of 30 × 30 cm^2^, positioned on the patient couch with the mattress removed. The thickness of the PMMA block, the distances between the x-ray focal spot and phantom and the x-ray beam area were systematically varied. Cylinder-shaped, test objects were positioned on top of the PMMA block ca 10 cm away from the image detector. Figure 1a shows a schematic view of the imaging geometry used with the fixed fluoroscopy system (Setup 1).

In the quality control measurements, the PMMA slab was replaced by a 2 mm thick copper filter (99.9% Cu, Cambridge Ltd, Huntingdon, UK) placed outside of the collimator housing (Figure 1b). The test object was positioned in the center of the beam on the image detector in order to obtain an easily reproducible imaging condition with a mobile C-arm (Setup 2). Hence, the measurement in Setup 2 is done with minimal intervention and magnification and therefore with limited influence of the focal spot size. This setup is more easily reproduced and do not involve a heavy PMMA block and patient couch. Acquisition modes, imaging parameters, contrast details and automatic dose rate control (ADRC) parameters for the two measurement setups are given in Table 1.

**Figure 3:**
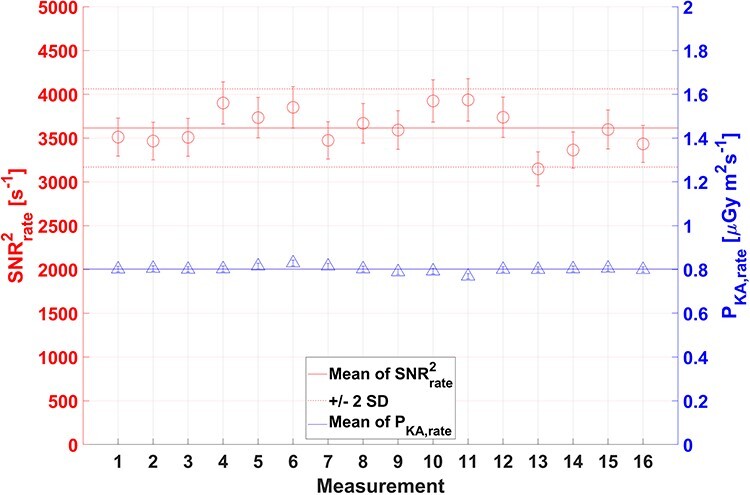
Repeated P_KA,rate_ and SNR^2^_rate_ measurements using Setup 2 with a Siemens Cios Alpha mobile C-arm over a period of 4 months. The contrasting detail was a 4 mm thick and 6 mm diameter Al-cylinder. The solid lines indicate the mean value and the dashed line indicate ±2 SDs (or 14%) in SNR^2^_rate_

### Uncertainty estimation

The relative uncertainty in SNR^2^_rate_ for different experiments was estimated to 7.1–9.4% by:

\begin{align*} {\sigma}_{\mathrm{SN}{\mathrm{R}}_{\mathrm{rate}}^2} =\sqrt{\sigma_{\mathrm{stat}}^2+{\left({\mathrm{B}}_{\mathrm{D}}\mathrm{D}{\sigma}_{\mathrm{rel},\mathrm{D}}\right)}^2+{\left({\mathrm{B}}_{{\mathrm{M}}^2}{\mathrm{M}}^2{\sigma}_{\mathrm{rel},{\mathrm{M}}^2}\right)}^2+{\left({\mathrm{B}}_{\mathrm{FS}}\mathrm{FS}{\sigma}_{\mathrm{rel},\mathrm{FS}}\right)}^2}, \end{align*}

where ${\sigma}_{\mathrm{Stat}}$ is the statistical uncertainty in the image analysis due to a limited number of image samples, estimated to 6–8% (see Figure 2b). A quadratic uncertainty term was then added for an experiment when a parameter X was altered between setups. ${\sigma}_{\mathrm{rel},\mathrm{X}}$ is the relative uncertainty in X and ${\mathrm{B}}_{\mathrm{x}}$ the slope of its linear relation with SNR^2^_rate_. A 1 cm display uncertainty in couch height results in a change in magnification (M) of the contrast detail (which affect SNR^2^_rate_) and ${\sigma}_{\mathrm{rel},{\mathrm{M}}^2}$ was estimated to 1.6–2.0%. The uncertainty in the measurements of x-ray field size ${\sigma}_{\mathrm{rel},\mathrm{FS}}$ is 2.4–4.6%.

The variation in dose index between subsequent measurements was estimated to 2% ${\sigma}_{\mathrm{rel},\mathrm{D}}$ from the spread of P_KA_-rate readings acquired in Setup 2. The accuracy in the calibration of the instruments PDC (P_KA_-meter, Radcal, Monrovia USA) and T20 (air kerma meter, RTI Group, Mölndal Sweden) were 2.4% (k = 2) and 1.7% (k = 2), respectively. The uncertainty in the figures of merit FOM_KA_ and FOM_K_ was estimated to 7.9–9.4% in Setup 1 experiments, where dose indices and SNR^2^_rate_ were treated as independent variables.

## RESULTS

### Software validation measurements

Figure 2a shows the influence on SNR^2^_rate_ of the area of a 3 mm thick Al cylindrical disc contrast detail using Setup 1. Figure 2b shows a linear increase of the relative statistical uncertainty in SNR^2^_rate_ when plotted against the inverse of the square root of the number of image frames used in the analysis. Using 1000 frames, the uncertainty (1 standard deviation, SD) in SNR^2^_rate_ is ca 7%. Figure 2c shows the linear increase in SNR^2^_rate_ with increasing tube current as indicated by the K_rate_ measured at the image detector (Setup 2).

### Quality control of key performance parameters

Figure 3 shows results of 16 repeated measurements of SNR^2^_rate_ and P_KA,rate_ over 4 months. The SD in SNR^2^_rate_ from repeated measurements was 6%. The results indicate that the imaging system was stable.

### Measurements of FOM

**Figure 4:**
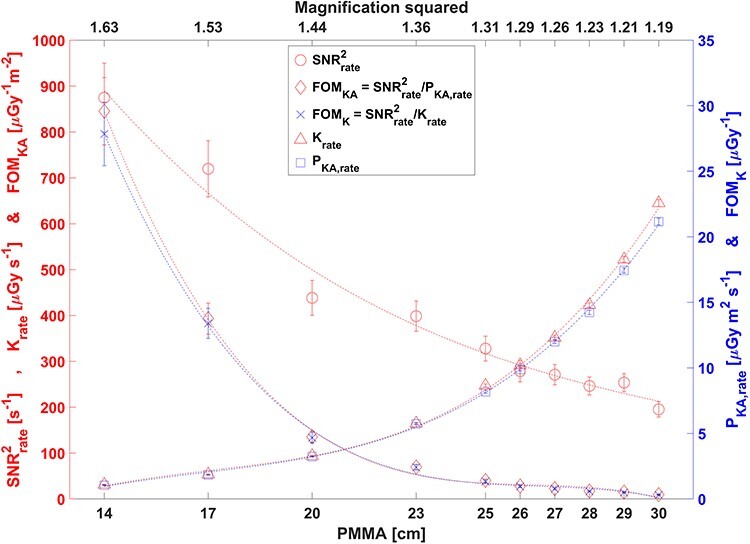
Image system characteristics for 10 different PMMA thicknesses from 14 to 30 cm with imaging conditions in Setup 1, but source image detector distance SID of 120 cm. With increasing PMMA thickness the tube current (20, 41, 83, 95, 97, 97, 97, 98, 101, 123 mA) and pulse length (3.5, 3.5, 3.4, 5.5, 7.8, 9.5, 11.6, 13.7, 16.3, 16.3 ms) increased while tube voltage and filtration were maintained (81 kV, 0.3 mm Cu filtration). The geometric magnification decreased as PMMA thickness increased. The fitted curves are not based on any model, but connect the data points to make the results more discernible

**Figure 5:**
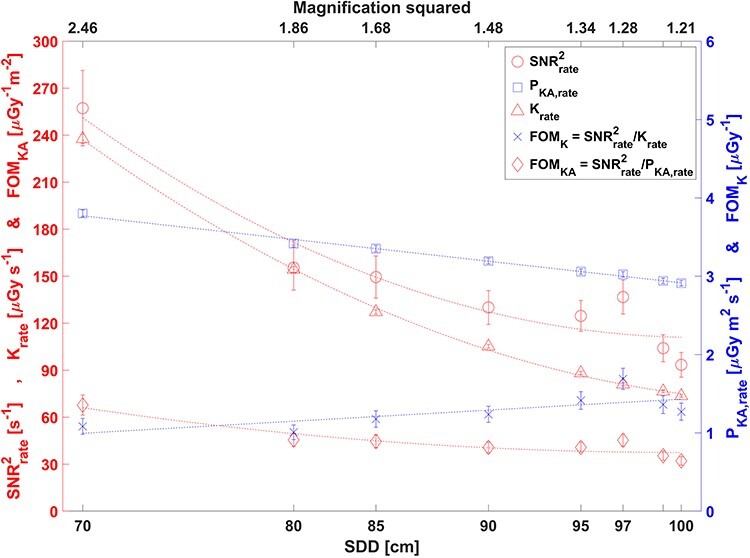
Image system characteristics for eight different source-to-detail distances, SDD with decreasing tube current (79, 70, 69, 66, 63, 62, 60, 60 mA) and image magnification, but constant pulse length, 81 kV, 0.3 mm Cu filtration and other imaging conditions as in Setup 1

Figures 4–5 and Table 2 show SNR^2^_rate_, K_rate_, P_KA,rate_, SNR^2^_rate_/K_rate_ and SNR^2^_rate_/P_KA,rate_ as a function of PMMA phantom thickness (Figure 4), source to contrast detail distance (Figure 5) and x-ray field size (Table 2). The results were expected and consistent with our experiences. The changes were due to the specific way the ADRC system was designed to approximately maintain air kerma rate at the image detector surface behind the anti-scatter grid.

## DISCUSSION

The main finding in this study was that using a model observer to assess an image quality index, such as SNR^2^_rate_, allows you to estimate small changes in the performance of the imaging system with high precision (6%). This is an advantage for quality control or for selecting a more dose efficient imaging setting.

Good agreement of SNR^2^_rate_ (within 1%) between results generated from the original FluoroQuality software^(7)^ and the in-house, MATLAB™-based version^(15)^ was found using identical image sets (Figure 2a). SNR^2^_rate_ increases linearly with both area of the contrast detail (for fixed K_rate_) and with K_rate_ (for fixed area contrast detail, A) in agreement with the so-called Rose-model, ${\mathrm{SNR}}_{\mathrm{rate}}^2\propto{\mathrm{M}}^2{\mathrm{C}}^2\mathrm{A}\ {\mathrm{K}}_{\mathrm{rate}}$, with C being the contrast and M the magnification.

We argue that the general trends of the variation of image quality index and dosimetric indices in Figures 4–5 and Table 2 are useful for teaching x-ray fluoroscopy physics and technology for clinical medical staff. They can be taught, discussed and reflected on during radiological protection training sessions with clinical staff. In fact, these and similar results are being used in training of resident radiologists in Linköping, Sweden. Tesselaar and Sandborg^(11)^ evaluated the figures of merit of changing the dose rate, pulse rate and field of view on a Siemens Axiom Artis Zee MP. In the present study, we assessed the figures of merit of the same equipment while instead changing the phantom thickness, x-ray field size and patient couch height. The results in terms of variation of SNR^2^_rate_, K_rate_, P_KA,rate_, SNR^2^_rate_/K_rate_ and SNR^2^_rate_/P_KA,rate_ with the imaging parameters above were expected, but specific to this imaging system and its ADRC-settings.

The large increase in K_rate_ and P_KA,rate_ with increasing PMMA thickness is evident in Figure 4 for fixed x-ray beam size and couch height. Both dosimetric indices approximately doubled for every additional 4 cm PMMA. The tube current initially increased with increasing PMMA thickness from 14 to 20 cm, whereas pulse length was approximately maintained. As the PMMA slab thickness was further increased, the pulse length increased while tube current was approximately maintained. SNR^2^_rate_ decreased rapidly with increasing PMMA thickness due to beam hardening and additional scatter to the image detector. The reduction in SNR^2^_rate_ was furthermore caused by a reduced magnification of the contrast detail (5 mm thick soft tissue), as it was positioned even closer to the image detector since the couch height was fixed while PMMA slab thickness increased. Consequently SNR^2^_rate_/K_rate_ and SNR^2^_rate_/P_KA,rate_ decreased at an equally rapid rate with increasing PMMA thickness.

As the source to detail distance (SDD) increased (by increasing the couch height; see Figure 1), the tube current decreased since more scattered radiation contributed to the ADRC (Figure 5). Source to image detector distance (SID), x-ray beam size, PMMA thickness and tube voltage were constant. Consequently K_rate_ and P_KA,rate_ also decreased, but K_rate_ decreased more rapidly with increasing SDD due to the inverse square law. SNR^2^_rate_ decreased with increasing SDD due to a decrease in magnification of the 15 mm thick low-density low contrast detail, lower photon fluence (decreasing tube current) and more scattered photons reaching the ADRC. FOM SNR^2^_rate_/P_KA,rate_ decreased slowly with increasing SDD since SNR^2^_rate_ decreased more rapidly than P_KA,rate_. SNR^2^_rate_/K_rate_, on the other hand, increased slowly with increasing SDD since K_rate_ decreased more rapidly than SNR^2^_rate_.

In Table 2, the ADRC-system responded to an increased amount of scattered radiation from an extended x-ray field size by decreasing the tube current. K_rate_ therefore decreased slowly. Magnification of the 5 mm thick soft tissue contrast detail was maintained and so was the PMMA phantom thickness. Since the increase in x-ray field size was much larger than the decrease in tube current, the P_KA,rate_ increased rapidly with x-ray field size. The reduced SNR^2^_rate_ was caused by an added proportion of scattered radiation and reduced K_rate_. SNR^2^_rate_/K_rate_ varied slowly with x-ray field size. However, SNR^2^_rate_/P_KA,rate_ decreased rapidly since SNR^2^_rate_ and P_KA,rate_ changed in opposite directions.

Previous studies have used model observers for the assessment of image quality in fluoroscopy systems. Bertolini^(16)^ used the Channelised Hotelling Observer model to assess possible significant differences between different imaging parameters on a General Electric (Discovery IGS740) fluoroscopy system. Their experiment is similar to the current study as it identifies imaging conditions with superior low-contrast detectability on a homogeneous phantom.

Villa^(6)^ developed a model observer approach to assess low-contrast detectability in dynamic imaging. In addition, they performed human observer performance assessments in the form of two -alternative forced-choice experiments and compared them with tuned model observers to identify best correlation. In contrast to our study, they did not explicitly compute a FOM nor attempt to use their image quality metric to quantify the quality of the specific angiography unit over time.

Samei^(17)^ pointed out the importance of anatomical background for the detection of lung nodules by human observers. He quantified its importance, as the much larger peak contrast-diameter product needed to detect nodules in an anatomical varying background compared to in a homogeneous background (with only quantum noise), for achieving identical area under the receiver operating characteristic curve (ROC-curve). This aspect is overlooked in our work. Therefore, general trends of figures of merit in our work need to be validated in a more realistic scenario with anatomical background and using model observers tuned to the human visual system.

Assessing low-contrast resolution with a human observer is quick, but probably biased and imprecise as humans find it difficult to define and reliably maintain what is actually resolved. In order to detect small changes in low-contrast resolution, we argue that a model observer will produce results that are more reliable. The sensitivity of SNR^2^_rate_ to detect changes in image noise is several times better than visual methods if one is limited to a reasonable number of human observers^(5)^. We find it useful not only to evaluate the image quality index SNR^2^_rate_ but also to measure simultaneously a dose rate index (e.g. P_KA,rate_ or K_rate_) to ensure that the ADRC-system is operating as expected.

The disadvantage of the SNR^2^_rate_ method is that it does not consider moving test objects and hence the effect of pulse length nor does it fully include the effect of the focal spot unsharpness if the test object is directly on top of the image detector housing. Moreover, a single type of test object may not be representative of all clinical tasks for which the system is used. The practical disadvantage of this model observer implementation is that it can be time-consuming (typically 10–15 minutes) to extract manually and analyses the images. However, if the images can be sent to a server and analyzed automatically when imaging is completed, the extra time is not a concern.

Dehairs^(18)^ implemented a spatio-temporal FOM [SdNR(u)] with a new ADRC strategy in dynamic imaging aiming to maintain the signal-to-noise level for a range of patient thicknesses. Contrary to what is found, for example in our Figure 4, using a conventional ADRC-system (where SNR^2^_rate_ decreases with increasing phantom thickness), their ADRC strategy keeps signal-to-noise constant from ~10 cm to 25 cm tissue-bone equivalent thickness and still results in an increase in their FOM, SdNR(u)^2^/AKR_ref_ compared with conventional ADRC; AKR_ref_ being the air kerma rate at the reference point. In effect, this new ADRC strategy adds an additional sixth parameter, the target detectability SdNR(u), to the traditionally used five parameters (tube voltage, tube current, pulse length, filtration and focal spot size).

## CONCLUSION

We have successfully implemented the FluoroQuality computer program in MATLAB™. The precision in the estimation of SNR^2^_rate_ in quality control is 6%. Our estimation of SNR^2^_rate_ or of FOM (e.g. SNR^2^_rate_/K_rate_ and SNR^2^_rate_/P_KA,rate_) allows staff to identify small but important improvements. The objective nature of the data provides reliable and transportable information for quality control and for teaching radiological protection to clinical staff.
